# Institutional Surgical Response and Associated Volume Trends Throughout the COVID-19 Pandemic and Postvaccination Recovery Period

**DOI:** 10.1001/jamanetworkopen.2022.27443

**Published:** 2022-08-18

**Authors:** Soham Ghoshal, Grant Rigney, Debby Cheng, Ryan Brumit, Michael S. Gee, Richard A. Hodin, Keith D. Lillemoe, Wilton C. Levine, Marc D. Succi

**Affiliations:** 1 Harvard Medical School, Boston, Massachusetts; 2 Medically Engineered Solutions in Healthcare Incubator, Innovation in Operations Research Center, Massachusetts General Hospital, Boston; 3 Department of Anesthesia, Massachusetts General Hospital Boston; 4 Department of Radiology, Massachusetts General Hospital, Boston; 5 Department of Surgery, Massachusetts General Hospital, Boston

## Abstract

**Importance:**

The COVID-19 pandemic is associated with decreased surgical procedure volumes, but existing studies have not investigated this association beyond the end of 2020, analyzed changes during the post–vaccine release period, or quantified these changes by patient acuity.

**Objective:**

To quantify changes in the volume of surgical procedures at a 1017-bed academic quaternary care center from January 6, 2019, to December 31, 2021.

**Design, Setting, and Participants:**

In this cohort study, 129 596 surgical procedure volumes were retrospectively analyzed during 4 periods: pre–COVID-19 (January 6, 2019, to January 4, 2020), COVID-19 peak (March 15, 2020, to May 2, 2020), post–COVID-19 peak (May 3, 2020, to January 2, 2021), and post–vaccine release (January 3, 2021, to December 31, 2021). Surgery volumes were analyzed by subspecialty and case class (elective, emergent, nonurgent, urgent). Statistical analysis was by autoregressive integrated moving average modeling.

**Main Outcomes and Measures:**

The primary outcome of this study was the change in weekly surgical procedure volume across the 4 COVID-19 periods.

**Results:**

A total of 129 596 records of surgical procedures were reviewed. During the COVID-19 peak, overall weekly surgical procedure volumes (mean [SD] procedures per week, 406.00 [171.45]; 95% CI, 234.56-577.46) declined 44.6% from pre–COVID-19 levels (mean [SD] procedures per week, 732.37 [12.70]; 95% CI, 719.67-745.08; *P* < .001). This weekly volume decrease occurred across all surgical subspecialties. During the post–COVID peak period, overall weekly surgical volumes (mean [SD] procedures per week, 624.31 [142.45]; 95% CI, 481.85-766.76) recovered to only 85.8% of pre–COVID peak volumes (*P* < .001). This insufficient recovery was inconsistent across subspecialties and case classes. During the post–vaccine release period, although some subspecialties experienced recovery to pre–COVID-19 volumes, others continued to experience declines.

**Conclusions and Relevance:**

This quaternary care institution effectively responded to the pressures of the COVID-19 pandemic by substantially decreasing surgical procedure volumes during the peak of the pandemic. However, overall surgical procedure volumes did not fully recover to pre–COVID-19 levels well into 2021, with inconsistent recovery rates across subspecialties and case classes. These declines suggest that delays in surgical procedures may result in potentially higher morbidity rates in the future. The differential recovery rates across subspecialties may inform institutional focus for future operational recovery.

## Key Points

**Question:**

How did surgical volumes change with respect to subspecialty and patient acuity during the COVID-19 pandemic, and did they recover after the peak and vaccine release periods?

**Findings:**

In this cohort study, a retrospective analysis of 129 956 records of weekly surgical procedures from January 6, 2019, to December 31, 2021, revealed that the overall volume did not fully recover to pre–COVID-19 levels well into 2021. Recovery rates were inconsistent across surgical subspecialties and case classes.

**Meaning:**

Further research and hospital-level changes are needed to address the backlog of surgical services and muted recovery of surgical procedures to pre–COVID-19 volumes.

## Teaser

This cohort study uses single-institution records to compare overall and subspecialty surgical volume trends before, during, and after the COVID-19 peak periods and after vaccine release.

## Introduction

The SARS-CoV2 pandemic has had an unprecedented impact on health care workers and the medical system. In following the US government’s recommendations to defer nonessential surgical care, many institutions made the decision to temporarily cancel elective surgery procedures to slow the spread of the virus.^1^ However, deferrals of certain elective surgeries have led to large backlogs in procedural care.^2^ Studies have estimated that these deferrals will grow to a cumulative backlog of more than 1 million cases 2 years after the end of elective surgery deferment.^3^ The pandemic also is associated with a reduction in surgical care for various racial and ethnic groups.^4^ Furthermore, there have been stark declines in both ambulatory care and necessary outpatient care visits, which other researchers have suggested may result in the development of more advanced disease states in the coming months or years.^5,6,7,8,9^ Other specialties, such as radiology, have experienced a significant reduction in imaging volumes with inconsistent recovery, including reduced emergency department diagnoses and initial cancer workups.^10,11,12,13,14,15^

To effectively minimize the impact of COVID-19 on surgical procedure volumes, our institution developed a phased approach to reductions in the number of operating rooms in line with the American College of Surgery’s guidelines for triage of nonemergent surgery.^16^ Incremental reductions in the number of operating rooms running helped to maintain capacity limits and created visual guidance for where nondeferrable cases should be placed. This response was necessary to meet the needs of the hospital’s patients as well as to balance the evolving needs of the hospital system.

A recent study showed that by the end of 2020, surgical volume across the US returned to 2019 rates in all surgical specialties except otolaryngology.^17^ However, despite having resumed elective cases, many hospitals were still operating at a substantially reduced capacity in 2021 and had significantly reduced imaging volumes.^15^ Furthermore, reductions in new diagnoses and emergent care likely have reduced sources of surgical procedures and bookings. Because many hospitals are now dealing with evolving variants, many surgery departments are again faced with altering operations and deferring elective procedures. A better understanding of how institutional policy changes are associated with surgical procedure volumes may guide institutional responses in the future. However, to our knowledge, this association has not been quantified, nor have surgical procedure volumes post–vaccine distribution been analyzed.

The present study aims to address this gap in the literature by quantifying the changes in surgery volume at a large academic quaternary care center in the northeastern US from the prepandemic period to December 2021. This may provide valuable insight into key disciplines that have recovered to comparable prepandemic rates and may inform institutional- and department-level focus for future pandemics or emergency situations.

## Methods

### Study Design and Setting

In this cohort study, we conducted a retrospective time-series analysis of all surgical procedures performed at Massachusetts General Hospital, a large (1017-bed) urban academic quaternary care institution, between January 6, 2019, and December 31, 2021. This study followed the Strengthening the Reporting of Observational Studies in Epidemiology (STROBE) reporting guideline for cohort studies.

This study was a single-institution, retrospective collection of aggregate data that was compliant with the Health Insurance Portability and Accountability Act and thus approved with exemption as secondary research for which consent is not required by the hospital’s institutional review board. Our main institution is a 1017-bed urban quaternary academic center that sees approximately 50 000 inpatients, 110 000 emergency department patients, and 1.5 million outpatients and conducts more than 20 000 operations per year.

This study included all surgical procedures conducted during the study period. Data regarding each individual procedure performed at our institution were extracted from the electronic health record (Epic Systems), yielding 129 956 entries during the 36-month study period. The surgical specialty for a given procedure was classified in the electronic health record by the clinician who performed the procedure. Completed procedures were first identified by filtering for entries that were labeled as completed. Individual entries were excluded if their procedures were labeled as canceled, voided, or scheduled.

### Variables

**Figure 1.**
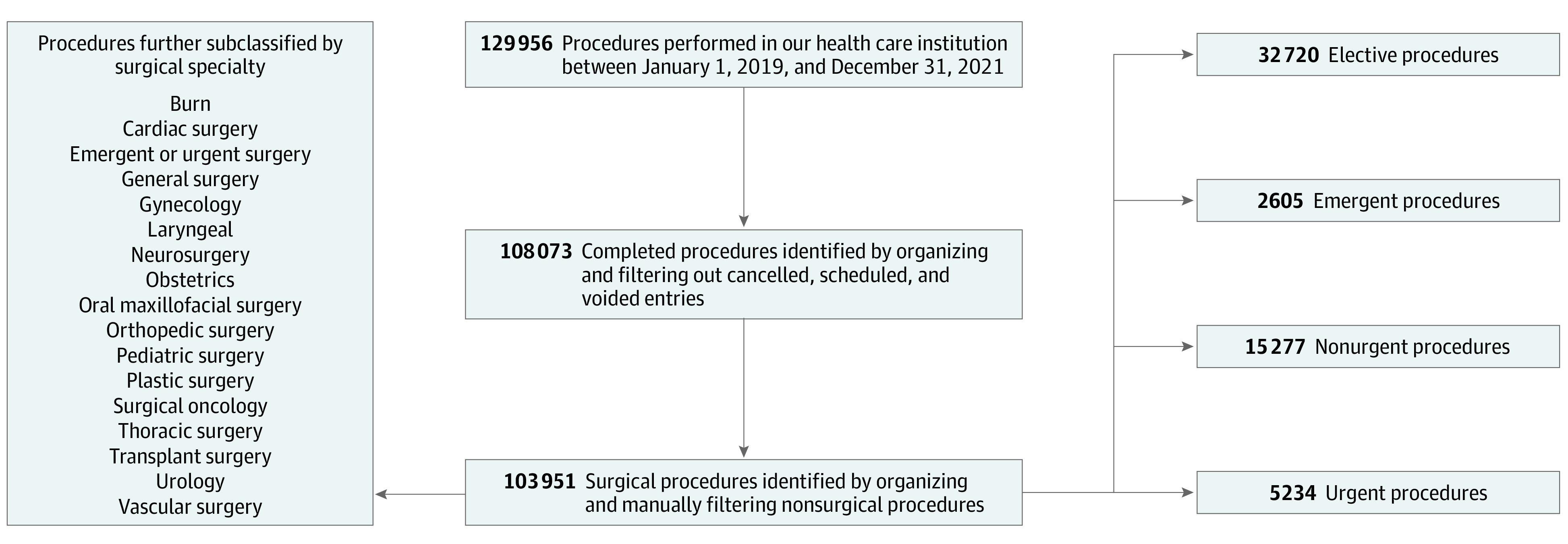
Flowchart Depicting Data Sorting and the Data Analysis Algorithm

The resulting 108 073 entries were subsequently screened to include subspecialties involving surgical procedures (burn, cardiac surgery, emergent or urgent surgery, general surgery, gynecology, laryngeal surgery, neurosurgery, oral maxillofacial surgery, orthopedic surgery, pediatric surgery, plastic surgery, surgical oncology, thoracic surgery, transplant surgery, urology, and vascular surgery). Data were also sorted according to case class (elective, emergent, nonurgent, and urgent) (Figure 1). Details regarding Massachusetts public health information and legislation were obtained from state websites and used to designate 4 distinct periods for the study: pre–COVID-19 (January 6, 2019, to January 4, 2020), COVID-19 peak (March 15, 2020, to May 2, 2020), post–COVID-19 peak (May 3, 2020, to January 2, 2021), and post–vaccine release (January 3, 2021, to December 31, 2021). March 15, 2020, was the date the Massachusetts Department of Public Health (DPH) issued a directive that all nonessential surgeries be deferred. May 18, 2020, was the date the DPH put into effect its phased reopening plan, which happened to be on a weekday. To conduct our analysis on a weekly basis, the week of May 17 was included in the post–COVID-19 peak period. Vaccine distribution to nonmedical community members in our state began on December 28, 2020, which happened to be on a weekday. To conduct our analysis on a weekly basis, the post–vaccine release period begins the following week on January 3, 2021.

### Statistical Analysis

We first developed autoregressive integrated moving average (ARIMA) models for total surgical volume and by specialty and patient acuity type to assess fluctuations in surgical volume over time. The ARIMA model was chosen to account for seasonality and temporal effects. We then calculated means and SDs of weekly surgical procedure volumes for each of the 4 study periods. Weeks were defined as Sunday to Saturday. Weeks 1 to 52 in 2019 were considered to represent the pre–COVID-19 period, weeks 11 to 19 in 2020 were considered to represent the COVID-19 peak, weeks 20 to 52 in 2020 were considered to represent the post–COVID-19 period, and weeks 1 to 52 in 2021 were considered to represent the post–vaccine release period. Weekly surgical volumes for the COVID-19 peak, post–COVID-19, and post–vaccine release periods were compared with their respective weeks in 2019 and analyzed as a proportion of the 2019 baseline and using a 2-sample *t* test assuming unequal variances. The analysis was then performed for each surgical subspecialty included in the data set, as well as across case class types. ARIMA modeling was done using Python, version 3.8.12 (Python Software Foundation), and statistical analysis was performed using and Excel, version 16.63 (Microsoft Corporation). Statistical significance was set at a 2-sided *P* < .05.

## Results

### Procedure Characteristics

Our sample comprised 129 956 surgical procedure records from January 6, 2019, to December 31, 2021 (Figure 1). Of these procedures, 108 073 (83.2%) were identified as completed, of which 55 836 (51.7%) had been labeled as either elective, emergent, nonurgent, or urgent. Of these labeled procedures, 32 720 (58.6%) were elective, 2605 (4.7%) were emergent, 15 277 (27.4%) were nonurgent, and 5234 (9.4%) were urgent.

#### Institutional Response to State DPH Directives

**Figure 2.**
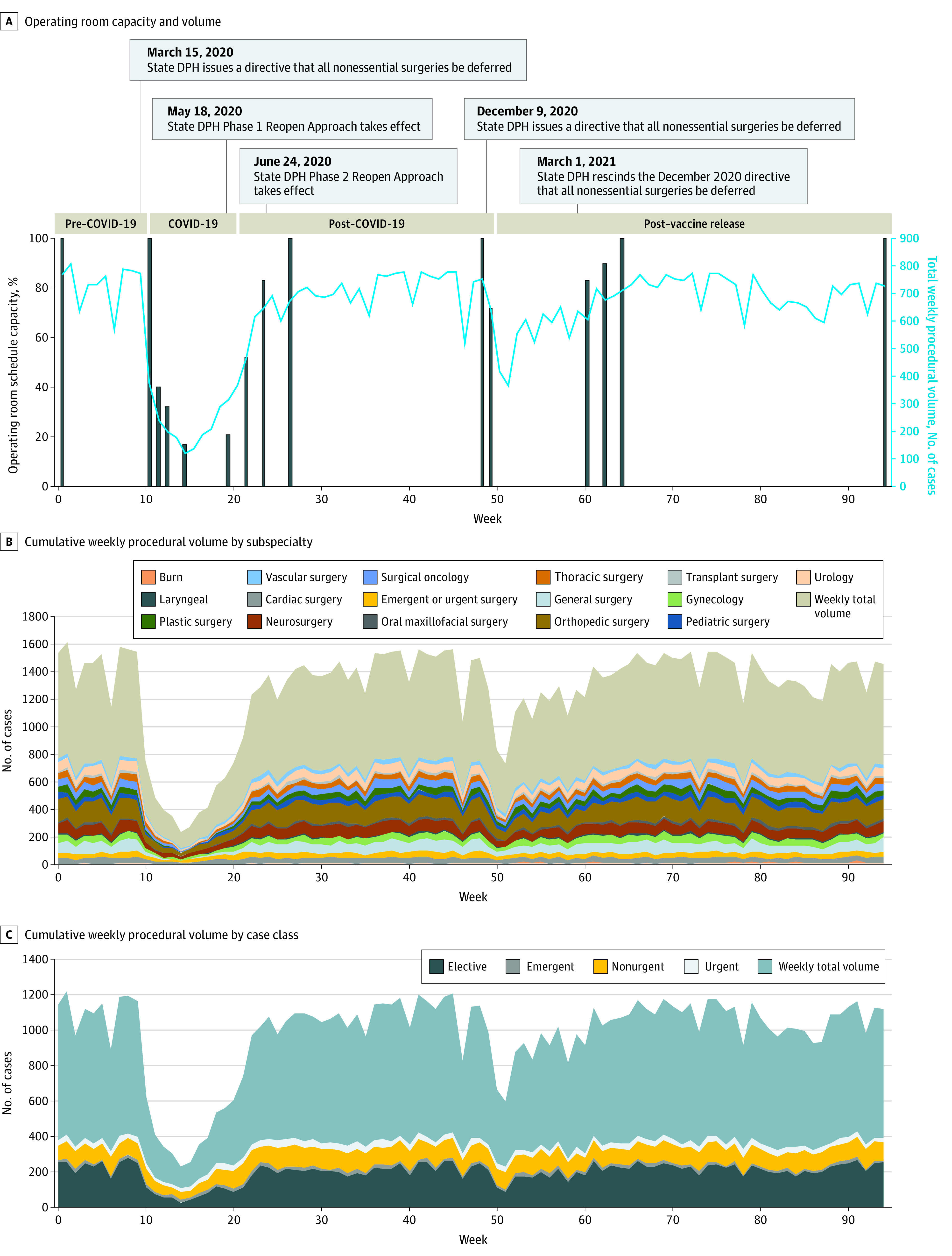
Timeline of Hospital Operating Room Schedule Capacity Limits in Response to State Department of Public Health (DPH) Nonessential Surgery Deferment Directives and Corresponding Procedural Volumes by Subspecialty and Case Class

After the declaration of a state of emergency, our hospital system developed a novel case deferment method in line with American College of Surgeons guidelines.^18^ Services were reallocated to conserve personal protective equipment, space, and staffing availability by quickly shifting operating room schedules. During the COVID-19 peak period, our hospital responded to the directive that all nonessential surgeries be deferred by reducing the hospital operating room capacity to 17% of the pre–COVID-19 capacity. During the post–COVID-19 peak period, when the state DPH issued a directive for hospitals to begin a phased reopening, our hospital subsequently increased operating room capacity to 83% of the pre–COVID-19 capacity. Finally, after the DPH phase 2 reopening directive on June 24, 2020, our hospital quickly recovered to full pre–COVID-19 operating room capacity (Figure 2).

In the week before the introduction of the phase 1 vaccine distribution plan, the state DPH again issued a directive that all nonessential surgeries be deferred in response to rising COVID-19 positivity rates. To accommodate this directive, our hospital reduced operating room capacity to 72% of prepandemic levels at the beginning of the post–vaccine release period. This directive was rescinded on March 1, 2021. Within a few weeks, our hospital returned to 100% operating room capacity (Figure 2).

#### Trends in Surgical Procedure Volumes During the COVID-19 Peak

After a state of emergency was declared in Massachusetts on March 10, 2020, peak COVID-19 surgical procedure volumes (mean [SD] weekly procedures, 406.00 [171.45]; 95% CI, 234.56-577.46) declined by 44.6% from pre–COVID-19 volumes (mean [SD] weekly procedures, 732.37 [12.70]; 95% CI, 719.67 to 745.08; *P* < .001) (Table 1 and Table 2).

**Table 1.** Descriptive Statistics of Overall 2019 Pre–COVID-19, 2020 Peak COVID-19, 2020 Post–COVID-19, and 2021 Post–Vaccine Release Period Weekly Surgical Procedure Volumes

Type of procedure	Weekly procedures, mean (SD)			
	Pre–COVID-19	COVID-19 peak	Post–COVID-19 peak	Post–vaccine release
All	732.37 (12.70)	406.00 (171.45)	624.31 (142.45)	672.55 (56.81)
By specialty				
Burn	12.30 (2.53)	7.02 (2.21)	11.08 (2.51)	14.09 (3.02)
Cardiac surgery	40.99 (4.62)	29.75 (6.93)	34.85 (4.94)	36.41 (4.22)
Emergent or urgent surgery	36.32 (3.62)	25.91 (5.03)	37.42 (3.93)	34.31 (2.14)
General surgery	72.34 (11.31)	29.65 (25.09)	63.21 (20.29)	66.08 (13.26)
Gynecology	49.44 (6.47)	24.69 (7.01)	39.48 (7.46)	47.94 (7.30)
Laryngeal	8.12 (2.47)	1.89 (0.04)	6.44 (2.35)	7.26 (2.37)
Neurosurgery	75.78 (6.85)	46.24 (10.11)	73.66 (11.85)	71.43 (6.58)
Oral maxillofacial surgery	15.45 (2.80)	4.52 (2.94)	12.91 (3.40)	15.04 (2.78)
Orthopedic surgery	142.56 (6.68)	71.77 (31.35)	125.16 (31.16)	133.84 (18.41)
Pediatric surgery	31.66 (4.48)	15.56 (6.82)	27.53 (6.91)	32.50 (5.55)
Plastic surgery	39.98 (5.58)	10.50 (11.62)	35.30 (10.58)	39.89 (6.79)
Surgical oncology	53.96 (7.64)	27.91 (14.54)	38.56 (12.85)	45.44 (6.30)
Thoracic surgery	38.59 (4.99)	21.50 (11.33)	33.37 (8.36)	35.63 (5.23)
Transplant surgery	15.25 (3.00)	7.08 (4.26)	12.61 (3.48)	14.75 (2.87)
Urology	64.49 (7.41)	31.23 (23.05)	52.11 (13.37)	52.72 (7.35)
Vascular surgery	26.80 (3.25)	16.09 (4.52)	24.91 (4.00)	24.66 (2.88)
By case class				
Elective	234.09 (30.98)	112.62 (55.80)	190.95 (46.88)	211.61 (29.79)
Emergent	17.08 (1.90)	16.98 (1.89)	17.30 (1.99)	16.32 (2.16)
Nonurgent	96.29 (3.74)	69.94 (15.68)	103.46 (10.25)	100.05 (4.05)
Urgent	36.63 (3.80)	27.13 (3.52)	34.58 (3.34)	30.81 (2.40)

**Table 2.** Relative Procedural Volumes in the COVID-19 Peak, Post–COVID-19 Peak, and Post–Vaccine Release Periods Compared With 2019 Baseline Volumes

Type of procedure	Volume during COVID-19 peak, % of baseline	*P* value	Volume during post–COVID-19 peak, % of baseline	*P* value	Volume during post–vaccine release, % of baseline	*P* value
All	55.4	<.001	85.8	<.001	92.9	<.001
By specialty						
Burn	52.3	<.001	94.0	.23	114.5	.001
Cardiac surgery	72.6	.001	88.5	<.001	88.8	<.001
Emergent or urgent surgery	73.8	<.001	98.4	.41	94.5	<.001
General surgery	40.3	<.001	86.6	<.001	91.3	.01
Gynecology	44.4	<.001	81.5	<.001	97.0	.27
Laryngeal	23.0	<.001	80.9	.02	89.5	.07
Neurosurgery	61.0	<.001	97.5	.43	94.3	.001
Oral maxillofacial surgery	31.2	<.001	80.5	<.001	97.4	.46
Orthopedic surgery	51.4	<.001	87.0	.002	93.9	.002
Pediatric surgery	49.0	<.001	85.2	.002	102.6	.40
Plastic surgery	26.1	<.001	87.9	.03	99.8	.94
Surgical oncology	50.2	<.001	72.2	<.001	84.2	<.001
Thoracic surgery	53.9	<.001	86.7	.004	92.3	.004
Transplant surgery	45.4	<.001	84.5	.003	96.7	.39
Urology	46.0	.001	82.4	<.001	81.8	<.001
Vascular surgery	55.3	<.001	95.4	.18	92.0	<.001
By case class						
Elective	44.1	<.001	85.0	<.001	90.4	<.001
Emergent	100.1	.99	101.3	.64	95.6	.06
Nonurgent	73.7	.001	107.5	<.001	103.9	<.001
Urgent	78.0	<.001	90.3	<.001	84.1	<.001

This weekly decrease was seen across all surgical subspecialties included, with the largest declines seen in laryngeal surgery (−77.0%; mean [SD] weekly procedures, 1.89 [0.04]; 95% CI, 1.85-1.93; *P* < .001), plastic surgery (−73.9%; mean [SD] weekly procedures, 10.50 [11.62]; 95% CI, –1.12 to 22.12; *P* < .001), oral maxillofacial surgery (−68.8%; mean [SD] weekly procedures, 4.52 [2.94]; 95% CI, 1.58-7.47; *P* < .001), and general surgery (−59.7%; mean [SD] weekly procedures, 29.65 [25.09]; 95% CI, 4.57-54.74; *P* < .001). Conversely, the least affected surgical subspecialties included emergent or urgent surgery (−26.2%; mean [SD] weekly procedures, 25.91 [5.03]; 95% CI, 20.88-30.95; *P* < .001) and cardiac surgery (−27.4%; mean [SD] weekly procedures, 29.75 [6.93]; 95% CI, 22.82-36.68; *P* = .001) (Table 1 and Table 2). During the COVID-19 peak period, weekly procedure volumes also significantly decreased across all case class types except emergent, which showed no change (Table 1).

#### Trends and Overall Recovery of Surgical Procedure Volumes in the Post–COVID-19 Peak Period

In the 7-month period after the peak of COVID-19, the overall volume of surgical procedures performed per week had not yet recovered to pre–COVID-19 volumes, reaching a mean (SD) of 624.31 (142.45) procedures (95% CI, 481.85-766.76 procedures) per week or 85.8% of the pre–COVID-19 baseline (*P* < .001) (Table 1). In addition, the recovery was inconsistent across subspecialties and case classes.

During the post–COVID-19 period, several specialties did not recover to their respective pre–COVID-19 baseline volumes. The specialties with the least recovery were surgical oncology (72.2% of baseline; mean [SD], 38.56 [12.85] procedures; 95% CI, 25.71-51.42 procedures; *P* < .001), oral maxillofacial surgery (80.5% of baseline; mean [SD], 12.91 [3.40] procedures; 95% CI, 9.52-16.31 procedures; *P* < .001), and laryngeal surgery (80.9% of baseline; mean [SD], 6.44 [2.35] procedures; 95% CI, 4.09-8.80 procedures; *P* = .02). However, emergent or urgent surgery (98.4% of baseline; mean [SD], 37.42 [3.93] procedures; 95% CI, 33.49-41.34 procedures; *P* = .41), neurosurgery (97.5% of baseline; mean [SD], 73.66 [11.85] procedures; 95% CI, 61.81-85.51 procedures; *P* = .43), and vascular surgery (95.4% of baseline; mean [SD], 24.91 [4.00] procedures; 95% CI, 20.91-28.91 procedures; *P* = .18) recovered to their respective pre–COVID-19 volumes.

Procedure recovery by case class followed a similar pattern, with only some case types recovering. Emergent procedures recovered to pre–COVID-19 volumes. However, elective procedure volumes recovered to only 85.0% of baseline (mean [SD], 190.95 [46.88] procedures; 95% CI, 144.07-237.82 procedures; *P* < .001), urgent procedures recovered to only 90.3% of baseline (mean [SD], 34.58 [3.34] procedures; 95% CI, 31.24-37.92 procedures; *P* < .001), and nonurgent procedure volumes recovered to 7.5% beyond baseline levels (mean [SD], 103.46 [10.25] procedures; 95% CI, 93.21-113.71 procedures; *P* < .001).

#### Trends and Recovery of Surgical Procedure Volumes in the Post–Vaccine Release Period

On December 15, 2020, phase 1 of the Massachusetts COVID-19 vaccine distribution plan began, allowing vaccinations for clinical and nonclinical health care workers participating in direct and COVID-19–facing care. This early phase of the COVID-19 vaccine distribution plan closely aligned with the second state DPH directive to defer nonessential surgical procedures in response to increasing COVID-19 burden and positivity rates. We conducted an analysis of surgical volume recovery during the 12 months following this important milestone in the pandemic. During this period, the mean (SD) overall volume of surgical procedures performed per week was 672.55 (56.81), which was still significantly lower than pre–COVID-19 baseline levels (95% CI, 615.74-729.36 procedures; 92.9% of baseline; *P* < .001). It was found that 5 of the subspecialties that did not recover procedural volumes in the post–COVID-19 peak period also did not experience a recovery to pre–COVID-19 volumes in the post–vaccine release period (Table 1). These subspecialties included cardiac surgery, urology, orthopedic surgery, surgical oncology, and thoracic surgery. Unexpectedly, although emergent or urgent surgery and vascular surgery had recovered their procedural volumes in the post–COVID-19 period, they experienced further significant declines in procedural volumes during the post–vaccine release period to 94.5% of baseline (mean [SD], 34.31 [2.14] procedures; 95% CI, 32.17-36.46 procedures; *P* < .001) and 92.0% of baseline (mean [SD], 24.66 [2.88] procedures, 95% CI, 21.78-27.54 procedures; *P* < .001), respectively.

The trends in the post–vaccine release period mimicked those of the post–COVID-19 peak period. Elective and urgent procedure volumes remained significantly lower than baseline, and nonurgent procedure volumes remained higher than baseline (Table 1).

## Discussion

This cohort study adds to the literature suggesting that COVID-19 severely impacted surgical care throughout 2020 as well as in 2021, despite broad vaccination efforts.^17,19,20^ To our knowledge, this study is the most comprehensive analysis to date of the association of COVID-19 with surgical procedure volumes by specialty and case type beyond 2020. The findings are in line with work that has revealed substantial declines in surgical care from 2019 to 2020, with declines that never recovered to prepandemic levels.^4^ In this study, we recapitulated these declines in surgical volume from the pre–COVID-19 period to the peak of the pandemic, with differential recoveries in the post–COVID-19 peak and post–vaccine release periods in 2021. Many of these reductions were driven by our hospital’s quick surgical case deferment policies, which empowered many different surgical specialties to safely and quickly reduce operating room schedules and capacity during the early weeks of the pandemic. Our results are consistent with previous clinical findings of dramatic declines in volumes of cardiac, orthopedic, otolaryngologic, and urgent or emergent vitreoretinal surgical procedures.^21,22,23,24^ Of note, these studies did not analyze surgery volumes past the end of 2020 and, thus, did not provide insight into the longer-term associations of the pandemic or vaccine release with surgical care. Our study addresses this gap in the literature by investigating surgical volumes during the start of the pandemic through December 2021.

After the Massachusetts DPH’s directive to defer all nonessential surgeries, our institution rapidly triaged nonemergent surgeries and reduced operating room capacity to 17% of prepandemic levels within a few weeks. Importantly, this response allowed staffing and personal protective equipment resources to be redirected to the care of the growing burden of COVID-19, particularly during the peak period.^16^ This deferment also allowed for operating room resources to be reallocated for life- and limb-saving procedures, treatment of unstable diseases, certain aggressive cancers, and severe acute symptoms and fractures.

We found that recovery trends differed across subspecialties and case classes. The recovery of different specialties’ surgical procedure volumes may be influenced by a variety of barriers in different surgical departments, such as workforce shortages, inpatient bed availability, operating room capacity, and operating room turnover times. These factors have been previously reported by providers as barriers to increasing surgery volumes.^2^

Finally, we found that during the post–vaccine release period, there was a full recovery of nonurgent procedure volumes. However, overall surgical procedure volumes remained significantly lower than pre–COVID-19 levels. In concordance, we found insufficient recovery in cardiac surgery, general surgery, urology, orthopedic surgery, surgical oncology, and thoracic surgery procedures. We speculate that, again, this insufficient recovery could be associated with department-specific differences in barriers to increasing surgery volumes, such as workforce shortages and facility capacities (eg, inpatient beds and operating rooms), and operating room turnover times. There may also be a lasting effect from reduced imaging volumes throughout the pandemic—for example, prior work has found reduced cancer screenings and initial diagnosis by imaging, which may reduce future surgery cases.^10^

These findings suggest that delays in surgeries may increase morbidity and mortality rates in the future. A body of studies has indicated that COVID-19 has caused the deferral of millions of elective procedures, resulting in a potentially large backlog of case volumes.^25^ In a survey of health system leaders, hospital executives reported that this backlog may be difficult to clear given workforce availability, enhanced sanitation protocols, and reserved inpatient capacity.^2^ This backlog may lead to increases in morbidity rates and deaths, as deferring needed treatment can have significant risks.^6^ For example, for patients awaiting coronary surgery, the median waiting list mortality rate is 2.6% per month, with mortality risk increasing at 11% per month. Furthermore, 12% of patients experienced a myocardial infarction while on the waiting list.^10,11^ To our knowledge, our study is the first to examine the widespread association of COVID-19 with surgical care approximately 2 years since the first wave.^26^ Thus, although the pandemic may subside in the coming months, our data reveal that health delivery systems in the US must prepare for a quickly advancing wave of surgical need.

### Limitations

This study has several key limitations. The data were gathered from 1 institution in the US, which limits its external validity. Surgery volume rates are influenced by many factors, including local and state COVID-19 policies, the degree of severity of COVID-19 infection in the community in which the hospital operates, and individual institutional decisions based on administrator judgment. Although the second state DPH directive coincided with the beginning of the vaccine rollout, vaccines were initially limited to certain populations, which may have affected COVID-19 positivity rates. In addition, this study evaluated surgery numbers in aggregate without considering patient factors that could influence surgery volumes apart from institutional factors. For instance, some patients may elect to defer surgery because they believe that the risks outweigh the benefits, even if the institution allows and has the capacity for said procedures. Despite these limitations, we believe that the overall trends in surgery volumes reflect broader national trends, possess some degree of external validity, and provide insight into which disciplines deserve specific efforts to return to normal productivity levels.

## Conclusions

In this cohort study using retrospective time-series analysis of surgical volumes during the COVID-19 pandemic, we have revealed a severe decline in surgical procedure volumes across a multitude of subspecialties during the COVID-19 peak, followed by an inconsistent and insufficient recovery for many specialty services during the 7-month post–COVID-19 peak period and 12-month post–vaccine release period. Future studies should aim to quantify the longer-term associations of the pandemic with surgical care by examining the impact of the Delta and the Omicron variants into 2022 and beyond.
